# 1828. Dalbavancin Utilization and Associated Clinical Outcomes: A Case-Series

**DOI:** 10.1093/ofid/ofac492.1458

**Published:** 2022-12-15

**Authors:** Meagan N Greckel, Brent Footer, Alyssa Christensen, Alyssa Christensen, Gregory B Tallman, Lea Edwards

**Affiliations:** Providence Saint Vincent Medical Center, Grand Junction, Colorado; Providence Portland Medical Center, Portland, Oregon; Providence Saint Vincent Medical Center, Grand Junction, Colorado; Providence Saint Vincent Medical Center, Grand Junction, Colorado; Pacific University School of Pharmacy, Portland, Oregon; Providence Saint Vincent Medical Center, Grand Junction, Colorado

## Abstract

**Background:**

Dalbavancin is an intravenous (IV) long acting lipoglycopeptide approved for use in acute bacterial skin and skin structure infections (ABSSSI). However, given the prolonged half-life, there is interest in using this agent as an alternative therapy for patients unable or unwilling to comply with conventional IV therapy. These include off-label treatment of deeper-seated infections such as infective endocarditis or osteomyelitis. This case series assesses the efficacy and safety of dalbavancin and its role as an alternative therapy in serious gram-positive infections.

**Methods:**

Retrospective case series of hospitalized adult patients who received at least one dose of dalbavancin at one of the eight hospitals within the Providence Oregon health system between December 2019 through January 2022. Eligible patients had appropriate follow-up within the 90-day period after the last dose was given. Patients who received dalbavancin as empiric therapy or had a vancomycin-resistant isolate were excluded. Clinical cure was defined as no clinical, laboratory, or microbiological evidence of persistent or recurring infection during the 90-day follow-up period.

**Results:**

110 patients were included in this analysis; 41% were IV drug (IVDU) users. The average age was 50.5 years, and the most common organism was Methicillin-sensitive *Staphylococcus aureus* (48.2%). Bacteremia occurred in 50% of the cases, and the most common infection site was osteomyelitis or native joint infections (31.8%). 30-day all-cause readmission, recurrence, and mortality occurred in 12.7% (2/110), 1.8% (14/110), and 0%, respectively. 30 to 90-day all-cause readmission, recurrence, and mortality occurred in 14.5% (16/110), 7.3% (8/110), and 1.8% (2/110), respectively. Adverse events occurred in 4.5% (5/110) of the cases, where infusion related reactions were most common. Presumed clinical cure occurred in 89% (98/110).

**Conclusion:**

A high percentage of patients in this case series were bacteremic, which differs from prior reports. Overall, dalbavancin appears to be a safe and effective treatment alternative for deeper-seated infections, including those with concomitant bacteremia.

**Disclosures:**

**All Authors**: No reported disclosures.

